# Source Apportionment of Heavy Metals Based on Multiple Approaches for a Proposed Subway Line in the Southeast Industrial District of Beijing, China

**DOI:** 10.3390/ijerph20010683

**Published:** 2022-12-30

**Authors:** Xiaoyang Jia, Tianxiang Xia, Jing Liang, Yandan Li, Xiaoying Zhu, Dan Zhang, Jinsheng Wang

**Affiliations:** 1 College of Water Sciences, Beijing Normal University, Beijing 100875, China; 2 Beijing Key Laboratory for Risk Modeling and Remediation of Contaminated Sites, Beijing Municipal Research Institute of Eco-Environmental Protection, Beijing 100037, China; 3 Advanced Institute of Natural Sciences, Beijing Normal University, Zhuhai 519087, China

**Keywords:** soil heavy metals, source apportionment, multiple approaches, positive matrix factor

## Abstract

Apportioning the sources of heavy metals (HMs) in soil is of great importance for pollution control. A total of 64 soil samples from 13 sample points at depths of 0–21 m were collected along a proposed subway line in the southeast industrial district of Beijing. The concentrations, distribution characteristics, and sources of eight HMs were investigated. The results showed that the concentrations of Hg, Cd, Cu, Pb, As, and Zn in the topsoil (0–2 m) exceeded the Beijing soil background values. Three sources were identified and their respective contribution rates calculated for each of the HMs using multiple approaches, including correlation analysis (CA), top enrichment factor (TEF), principal component analysis (PCA), and positive matrix factor (PMF) methods. As (63.11%), Cr (61.67%), and Ni (70.80%) mainly originated from natural sources; Hg (97.0%) was dominated by fossil fuel combustion and atmospheric deposition sources; and Zn (72.80%), Pb (69.75%), Cu (65.36%) and Cd (53.08%) were related to traffic sources. Multiple approaches were demonstrated to be effective for HM source apportionment in soil, whilst the results using PMF were clearer and more complete. This work could provide evidence for the selection of reasonable methods to deal with soils excavated during subway construction, avoiding the over-remediation of the soils with heavy metals coming from natural sources.

## 1. Introduction

Heavy metal pollution in urban soil has become an important threat to both the soil environment and human health in China [1,2,3,4]. This situation is more serious in the old industry districts because of the intense human activities [5,6,7]. In recent years, many old industry districts around the city have been shut down and relocated. Before site redevelopment, soil pollution status must be investigated, and corresponding risk control measures or remediation activities must be taken [8]. As is well known, HMs in the soil originate both from human activities (e.g., mineral resource development, metal processing and smelting, fossil fuel combustion, vehicle exhaust emissions, and wastewater irrigation), and from natural processes (e.g., weathering and pedogenesis processes, and precipitation) [9,10,11,12]. It is unreasonable and expensive to blindly carry out remediation activities regardless of the pollution sources. Thus, distinguishing various sources of HMs in soil is crucial for site remediation and environmental management.

The existing methods of analyzing sources of heavy metal pollution include geostatistical analysis, multivariate statistical analysis, and receptor model analysis methods. Geostatistical and multivariate analyses can only qualitatively identify the source of pollution [12,13,14]. Receptor models, such as chemical mass balance (CMB) [15,16,17], positive matrix factorization (PMF) [3,10,18,19,20], absolute principal component analysis–multivariate linear regression (APCA–MLR) [21,22,23,24], and the UNMIX model [25,26,27,28,29,30] can identify and quantify source contributions based on the chemical and physical characteristics of pollutants in sources and receptors. The CMB model is mainly used for source apportionment of atmospheric particulate matter [15,16,17]. The PMF model decomposes the original dataset into a contribution matrix and a profile group for contribution calculation and source identification [31], and has been widely used to determine the sources of pollutants in soil [3,10,18,19,20]. Guan et al. [18] used multivariate statistical analysis, including GIS-based geostatistical methods and PMF, to apportion sources of HMs in agricultural soil in the Hexi Corridor. Wu et al. [19] observed that the partition computing-based PMF approach can, to a certain extent, overcome the uncertainty of the spatial heterogeneity for source apportionment. APCA–MLR identifies the main pollution sources by reducing the dimension of the PCA data, and then determining the contribution rate of each pollution source using its absolute factor score and multiple linear regression of pollutant content [24]. Jin et al. [21] employed the APCA–MLR receptor model to identify pollution sources of heavy metals in farmland soil. Zhang et al. [22] suggested that the PMF approach could provide a more physically plausible source apportionment in the study area, and a more realistic representation of groundwater pollution, than the solutions from the APCA–MLR model. The UNMIX model was first applied to the source apportionment of atmospheric particulate matter [30], and has also been used to analyze heavy metal pollution sources in soil in recent years [25,26,27,28,29]. In addition, tracer techniques [32,33,34] and binding forms analysis [35,36] have also been applied to source apportionment. Wang et al. [32] revealed that the Bayesian stable isotope analysis mixing model (MixSIAR) could reduce the uncertainty of the PMF model and allocate the contributions of different sources more accurately. Sungur et al. [35,36] believe that HMs in uncontaminated soil are mainly immobile in forms bound to silicates and primary minerals, and that the anthropogenic and lithosphere sources of HMs are distinguished by analyzing different geochemical fractions. However, most of these studies are concerned with agricultural soil at a regional scale, and are mainly focused on the comparison of different approaches. The suitability of multiple approaches for source apportionment of soil pollution at site scale remains a challenge. Meanwhile, little attention has been devoted to the application of source apportionment results to site remediation and environmental management.

The Beijing southeast industrial district used to be one of the oldest industrial areas. Many highly polluting industries, such as coking and chemical plants, were scattered here. Severe pollution levels have been observed in this area [37,38]. According to the development plan, a new subway line will pass through this area. It is important to investigate the soil pollution conditions along the subway corridor and to identify the potential sources and their contributions to the soil contamination. The specific objectives of this study were (1) to investigate the content and distribution characteristics of eight HMs in this study area, (2) to explore the sources of different HMs using multiple approaches and to quantify the source contributions through PMF modeling, and (3) to provide a reference for soil remediation and environmental management of this subway line.

## 2. Materials and Methods

### 2.1. Study Area and Sample Collection

**Figure 1:**
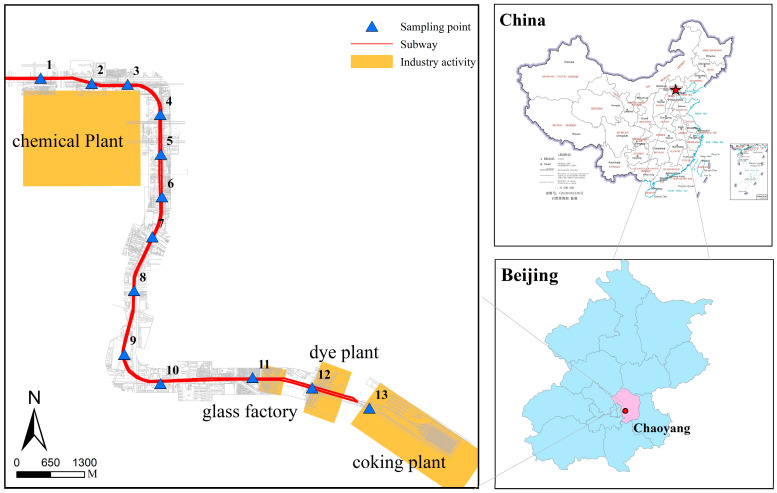
The location of the study area and distribution of 13 sampling points.

The proposed subway line, with a total length of 8.8 km, is located in the old chemical industrial area of Chaoyang district, Beijing (Figure 1). It consists of eight stations and seven depots. Many heavily polluting industrial enterprises were distributed along the subway corridor; including pharmaceutical, coking, chemical manufacturing, dyeing, and glassmaking plants, among others. All of these industrial enterprises have been shut down and relocated. Thirteen sampling points were chosen along the planned subway line, spaced as evenly as possible with an approximate interval of 600 m, as shown in Figure 1.

A total of 64 soil samples were collected, based on the pollution location and formation lithology, including 13 topsoil samples (0–2 m) and 51 subsoil samples (2–21 m). Soil profile samples were collected using a cable percussive drilling method. Percussive drilling makes use of the gravity and downward impulse of drilling tools to cause the drill to hit the bottom of the hole, so as to break up the soil layer and realize drilling. During drilling, the soil samples and cores taken by the core pipe were stored in a core box in sequence, and soil profile samples were taken from the fill layer (0.7–1.9 m), the upper weak permeable layer (2.2–6.6 m), the weak permeable layer (7.1–10.7 m), the aquifer (9.7–17.6 m), and the impermeable layer (16.6–20.9 m) within each borehole.

### 2.2. Sample Preparation and Analysis

All soil samples were naturally air-dried in a ventilated and dark laboratory, sieved into 60-mesh size particles after removing gravel, residual roots, and other unwanted materials, and then sealed in brown glass bottles and conserved in a refrigerator at 4 °C. As, Be, Cd, Cr, Cu, Ni, Pb, V, and Zn were analyzed using ICP-MS (Agilent 7500, Santa Clara, CA, USA). A 1.0 g aliquot of soil sample was weighed into a 50 mL polyethylene digestion tube, 4 mL (1 + 1) nitric acid and 10 mL (1 + 4) hydrochloric acid were added, and the mixture heated at 95 °C for 45 min. It was then slightly cooled and made up to volume with deionized water, shaken well and stood still before measurement.

Hg was determined using an atomic fluorescence photometer (AFS; Beijing Jitian Instruments Co., Ltd. Production, Beijing, China, AFS-820). A 0.250 g soil sample was weighed into a 25 mL colorimetric tube, 10 mL of aqua regia (HNO3:HCl = 3:1) was added, then it was shaken well and cold digested overnight. After placing in a boiling water bath for 2 h the next day, the volume was made up with deionized water, shaken well and stood still before measurement. In quality assurance and quality control (QA/QC) studies, the recovery of a spiked blank ranged from 80% to 112% within the control range of 80–120%, and the relative percentage differences (RPD) of duplicate samples ranged from 1% to 16% within the control range of 0–20%. The RPD of spike matrix duplicates was 0–16% within the control range of 0–20%. These results established that the precision and accuracy of our analytical method were acceptable.

### 2.3. Top Enrichment Factor

The TEF value is the concentration ratio between the topsoil and subsoil [12]. It is a method to differentiate the topsoil metals originating primarily from anthropogenic or natural sources. The TEF is calculated as per Equation (1) [13]:

(1) $$\mathrm{TEF}=\frac{C_{i\text{-}\mathrm{topsoil}}}{C_{i\text{-}\mathrm{subsoil}}}$$

where *C_i_*_-topsoil_ is the concentration of target metal *i* in topsoil, and *C_i_*_-subsoil_ is the concentration of target metal *i* in subsoil.

### 2.4. Principal Component Analysis

PCA was carried out to identify the HM sources. Varimax and Kaiser normalization rotation was applied because orthogonal rotation minimized the number of variables with a high loading of each component and facilitated the interpretation of results. The data analyses were performed using SPSS Statistics 22.0 (IBM Corp., Armonk, NY, USA). The biplot was drawn with OriginPro 2018C (OriginLab Corporation, Northampton, MA, USA).

### 2.5. Positive Matrix Factorization Method

PMF relies on a receptor model that quantifies the contribution of sources in samples based on the composition or fingerprints of the sources. In this study, PMF version 5.0 (U.S. EPA, Washington, DC, USA) was used for source apportionment. This model decomposes the original matrix *x_ij_* into two factor matrices, *g_ik_* and *f_kj_*, and a residual matrix *e_ij_*; the basic equation is as Equation (2):

(2) $$x_{ij}=\sum_{k=1}^{p}g_{ik}\cdot f_{kj}+e_{ij}$$

where *x_ij_* is the content of the *j*th HM in sample *i*; *g_ik_* is the contribution of the *k*th source for sample *i*; and *f_kj_* is the source profile of the *j*th HM in source *k*. The residual error matrix *e_ij_* is calculated as the minimum value of the objective function *Q*:

(3) $$Q={\sum_{i=1}^{n}\sum_{j=1}^{m}\left(\frac{e_{ij}}{u_{ij}}\right)}^{2}$$

where *u_ij_* refers to the uncertainty of the *j*th HM in *i* number of samples. The main feature of PMF is that the model requires the HM content of samples and the uncertainties of the content that are used to analyze the quality of the content values individually. The uncertainty can be calculated through various methods [39]. In this study, the uncertainty was calculated using Equations (4) and (5) as follows:

(4) $$\mathrm{If}\;{c_{\mathrm{ij}}}_{\;}\leq \;\mathrm{MDL},\;u_{ij}=\frac{5}{6}\times MDL$$

(5) $$\mathrm{If}\;{c_{\mathrm{ij}}}_{\;}{>}_{\;}\mathrm{MDL},\;u_{ij}=\sqrt{{\left(c_{ij}\times \theta \right)}^{2}+{\left(0.5\times MDL\right)}^{2}}$$

where *c_ij_* is the content of the element, mg/kg; *θ* is the error fraction; and MDL is the method detection limit.

## 3. Results

### 3.1. Descriptive Statistics Analysis

The basic descriptive analysis statistics are presented in Table 1.

For the topsoil, the average concentrations of HMs were in order of Zn (54.16 mg/kg) > Cu (23.74 mg/kg) > Pb (20.60 mg/kg) > Cr (19.40 mg/kg) > Ni (16.86 mg/kg) > As (7.52 mg/kg) > Hg (0.91 mg/kg) > Cd (0.09 mg/kg. The concentrations of Hg, Cd, Cu, Pb, As, and Zn exceeded the soil background values for Beijing [29], with the maxima exceeding these by multiples of 116.88, 2.15, 1.63, 1.12, 0.36, and 0.41, respectively.

For the subsoil, the average concentrations of HMs were in order of Zn (33.19 mg/kg) > Cr (16.66 mg/kg) > Ni (15.41 mg/kg) > Cu (11.98 mg/kg) > Pb (8.03 mg/kg) > As (5.94 mg/kg) > Cd (0.06 mg/kg) > Hg (0.04 mg/kg. Only Cd, As, Cu, and Hg exceeded the soil background values, with the maxima exceeding these by multiples of 1.12, 0.74, 0.19, and 1.96, respectively.

The coefficients of variation (CVs) also indicated that the regional differences in contaminant content of the topsoil (0.16–2.42, with low to high variation) were stronger than those in the subsoil (0.32–0.83, with median to high variation), especially for Hg. Therefore, the HM contamination in topsoil was heavier than that for the subsoil.

### 3.2. Distribution Characteristics

**Figure 2:**
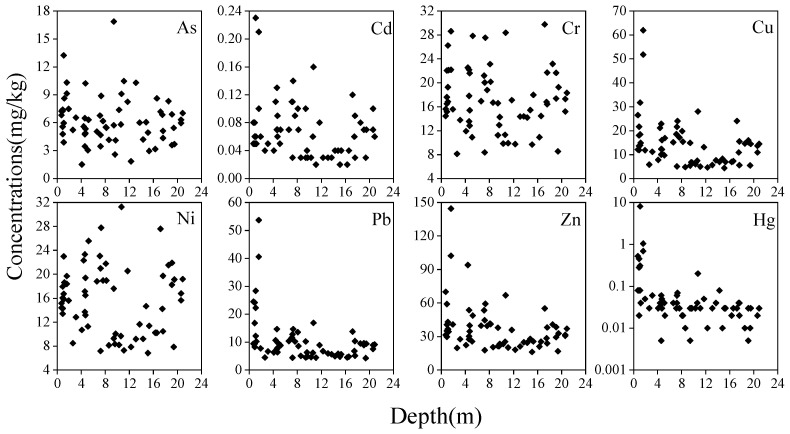
Vertical distributions of the HMs in the soil profile.

The vertical distributions of the eight HMs in the soil profile are presented in Figure 2. Concentrations of Cd, Cu, Pb, Zn, and Hg were significantly accumulated at the depth of 0–2 m, indicating that they had been affected by human activity [12]. The concentrations of Cr and Ni appeared to show no significant differences across the entire soil profile. As had higher concentrations at depths of approximately 10 m, which was exactly at the location of the groundwater level in this area. Accornero et al. [40] also found an accumulation of As in deep soil within the scope of our study area, and various geochemical processes were believed to be the main causes, such as the variation in soil composition, percentage and mineralogy of clays, organic carbon content, variation in the pH, exchange capacity of the soil, presence of either saturated or unsaturated layers, and compositional characteristics of the groundwater. Therefore, the HM content in the subsoil was unlikely to have been affected by human activity, and source apportionment was only carried out for the topsoil.

**Figure 3:**
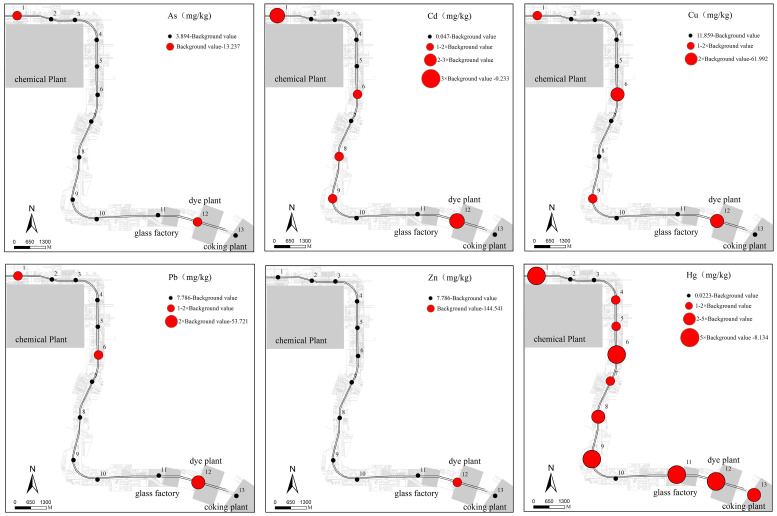
Horizontal distributions of the 6 excessive HMs in topsoil.

Figure 3 shows the horizontal distributions of the six HMs with contents exceeding the background values in the topsoil. For Hg, the samples showing concentrations higher than the background levels were evenly distributed across the study area, while those for the other five HMs were mainly concentrated at Points 1, 6, 9, and 12. Points 1, 6, and 12 were located near the former industrial enterprises, indicating that they might have been affected by production activities. However, Point 9 is far away from the production enterprises and located in the parking lot of a large playground. The organic carbon content in the topsoil was 15.3%, which was significantly higher than the average level (3.4%). Heavy metals in soils can complex and chelate with organic matter to fix them in soil [41], which might result in the enrichment of HMs at Point 9.

### 3.3. Source Apportionment

#### 3.3.1. Correlation Analysis

Spearman’s rank correlation coefficient was adopted to explore the relationships among the eight HMs in topsoil. As shown in Table 2, Cu, Pb, Zn, and Hg were significantly correlated with each other; Cr, Ni, and As were significantly correlated with each other; and Cd was significantly correlated both with As and with Cu, Pb, Zn, and Hg. The significant correlations indicate that these metals probably originated from the same source [12].

#### 3.3.2. Top Enrichment Factor Analysis

**Figure 4:**
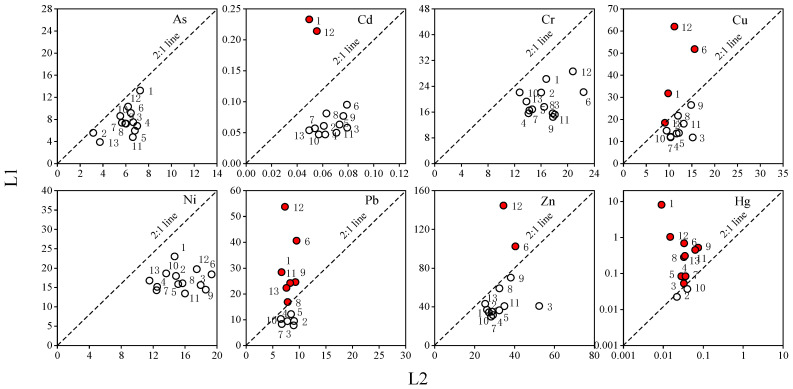
Concentrations of HMs in topsoil vs. subsoil.

As shown in Figure 4 and Table A1, the TEF values for As, Cr, and Ni were lower than 2 in all soil samples. Generally, a TEF value below 2 indicates a natural enrichment process [42]. A few sampling points for Cd, Cu, Pb, Zn, and Hg had TEF values greater than 2; mainly concentrated at Points 1 and 6 (both located near the chemical plant), and Point 12 (located near the dye plant), indicating that the topsoil might have been affected by anthropogenic processes [13].

#### 3.3.3. Principal Component Analysis

PCA was applied to analyze the sources of the HMs in topsoil. The first two principal components (PCs) explained 89.22% of the original information, with eigenvalues greater than 1 (Table A2).

**Figure 5:**
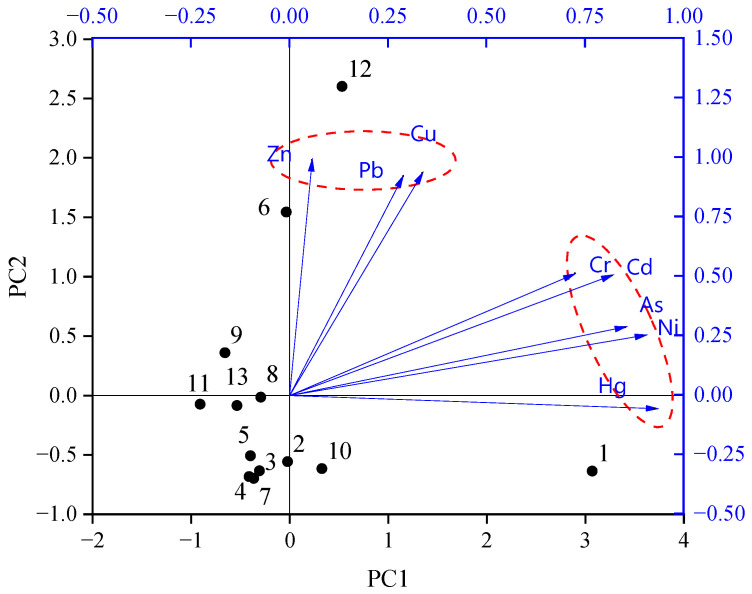
Biplots of sampling points and component 1 with component 2 based on the PCA results.

As shown in Figure 5 and Table A3, Hg (0.927), As (0.852), Ni (0.897), Cd (0.819), and Cr (0.725) had higher contributions to PC1, which was mainly expressed at Point 1—that located near the chemical plant. The concentrations of Hg, As, and Cd exceeded the background values in 76.92%, 15.38%, and 38.46% of the topsoil samples, respectively. However, Ni and Cr showed the opposite, and their TEF values were all below 2. Therefore, PC1 is considered to be related to mixed sources.

Zn (0.988), Cu (0.933), and Pb (0.920) had higher contributions to PC2, which was mainly expressed at Points 12 (located near the dye plant) and 6 (located near the chemical plant). The concentrations of Cu, Pb, and Zn exceeded the background values in 30.77%, 23.08% and 7.69% of the topsoil samples, respectively, and were significantly accumulated in the topsoil. Therefore, PC2 was considered to be related to anthropogenic source.

#### 3.3.4. Positive Matrix Factorization

**Figure 6:**
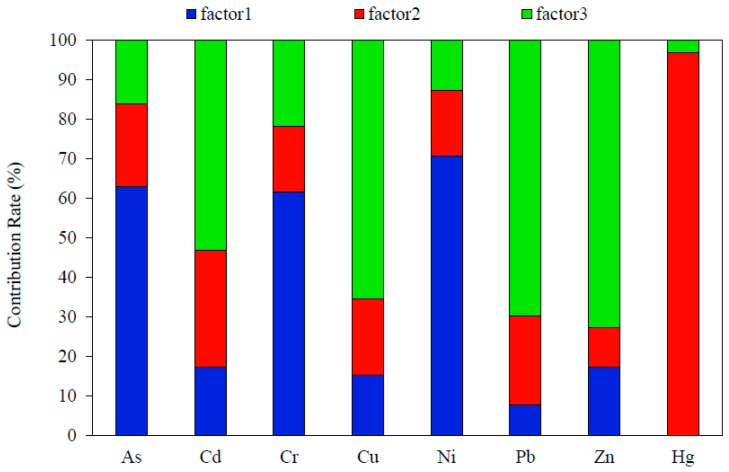
Contribution rates of the different sources of the HMs for topsoil.

The PMF model was employed to further determine and quantify the pollution sources and their contribution rates to the contaminants in the soils. The start seed number was chosen randomly, and the number of runs was set to 20. When the factor number was set to 3, the residual error was between −3 and 3, R2 was between 0.66 and 1.00, and the best fit results were obtained. Thus, the factor number was set to 3. The results of the factor profiles and source contributions of the HMs are shown in Figure 6.

The first factor accounted for 31.63% of the total variance, featuring high loadings of As (63.11%), Cr (61.67%), and Ni (70.80%). Many studies have reported that Cr, Ni, and As in soil would originate from soil parent materials [7,43,44]. Weathering processes of parent soil materials have been demonstrated to be a significant source for Cr, Ni, and As in both topsoil (0–20 cm) and subsoil (120–180 cm) in the Beijing urban area [45]. Sun et al. [7] found that parent materials and pedogenic processes were major factors in the levels and distribution of Ni, Cr, and As in soils in Tangshan. Ni and Cr are iron group elements; they can substitute for each other and create symbiosis enrichment in minerals [46]. Multiple studies have shown that Ni and Cr are derived from parent materials [47,48]. Accornero et al. [40] reported that the site-specific upper baseline concentration (UBC) of As in the study area is 10.4–12.6 mg/kg, which is higher than the wider scale background value for the Beijing area. In our study, As, Cr, and Ni were significantly correlated with each other, and their TEF values were all below 2. Only one sample had an As concentration (13.24 mg/kg) slightly greater than the UBC; this was located at a depth of 1.1 m at Point 1. It seems reasonable to attribute Factor 1 as a natural source.

The second factor accounted for 16.04% of the total variance, featuring high loading of Hg (97.0%). Similar results have been reported in which Hg exhibits a different pattern from the other HMs and is classified solely as a principal component in the source analysis [7,45,49,50]. About 1960 tons of Hg have been discharged to the environment every year from anthropogenic sources such as gold mining, coal combustion, and the production of non-ferrous metals [51,52]. Hg is a volatile component in coal; it is emitted in its oxidation state, particle state, and elemental state forms during the coal combustion process. The dust collector only had a high removal effect for the oxidation and particle state forms, while the elementary Hg would escape into the atmosphere and has entered into the soil through atmospheric transport and deposition [53,54]. A high mercury content caused by long-term coal combustion has also been confirmed in the urban soils of Beijing [55]. The area studied in this work was the earliest chemical industrial district in Beijing, with large-scale industrial enterprises such as coking and chemical plants which used coal as their main fuel, gathered in this region. The long-term production processes would inevitably lead to the escape and deposition of mercury. The topsoil in the studied area had a significantly enriched Hg content, with 76.92% of the topsoil samples having a Hg content exceeding the background value (Figure 2). Therefore, coal combustion was likely responsible for the enrichment of Hg in this area, and Factor 2 was attributed to the source of fossil fuel combustion and atmospheric deposition.

The third factor accounted for 52.33% of the total variance, featuring high loadings of Zn (72.80%), Pb (69.75%), Cu (65.36%), and Cd (53.08%). Previous studies have shown that soil Cd, Zn, Pb, and Hg levels are tightly associated with human activities [7,45,56,57,58]. Agricultural production and vehicular transport were the major factors in the concentrations of Cd, Zn, Pb, and Hg in soil from Shanxi Province [58]. van Bohemen [59] found that traffic emissions are the main source of Pb (fuel combustion), Zn (tire wear), and Cu (brake wear and radiator corrosion). Tetraethyl lead was used as an anti-knock agent in automobile gasoline; Pb would be emitted in the exhaust gas during the combustion process of gasoline, and enriched around the roadsides [49]. Zn was often used as a hardness additive in tires, and Cu was a component of brake pads [60]. High quantities of Cd and Zn have been observed in tire rubber [61]. Cd has been proven to be the primary pollutant of highway soil in Beijing, and has mainly come from motor vehicle exhaust and dust produced by tire wear and braking [56]. Zhang [62] also found high concentrations of Cd in smelter dust and automobile exhaust. In this study, Zn, Pb, Cu, and Cd were significantly enriched in the topsoil, and the soil boreholes were located along the main traffic roads, which indicates that traffic sources are major factors. Consequently, Factor 3 may represent traffic source.

#### 3.3.5. Comparison of Multiple Approaches

The source apportionment results obtained by multiple approaches were basically consistent. They all distinguished Cu, Pb, and Zn versus As, Cr, and Ni as two different sources, but were controversial in relation to Hg and Cd. CA classifies sources based on the degree of correlation between elements, but it was difficult to distinguish the elements with complex correlations. In this study, CA mixed the source of Cd with Cu, Pb, Zn, and As. TEF distinguishes natural and anthropogenic sources according to the accumulation of elements in topsoil, but it cannot subdivide anthropogenic sources and is not applicable to a situation where the subsoil contamination is heavier than the topsoil, such as contamination due to leakage of an underground tank. PCA and PMF are both factor analysis methods. PMF imposes non-negative limits on pollution sources and contributions, and also takes into account the uncertainties of the original data. Therefore, PMF can correct data deviations to make results more reliable. In this study, PCA failed to distinguish Hg and Cd from As, Cr, and Ni. PMF apportioned Hg as an isolated factor and classified Cd with Cu, Pb, and Zn as being from the same source—which agreed exceptionally well with existing research conclusions [10,36]. Overall, the results of CA and TEF were based on quantitative analysis, which could be used as a support for PCA and PMF, and the results of PMF were clearer and more complete compared to PCA.

## 4. Conclusions

The HM contamination status in the topsoil was heavier than that of the subsoil. The topsoil samples where Hg was over background levels were distributed evenly in the study area, but those for Cd, Cu, Pb, As, and Zn were mainly concentrated at Points 1, 6, 9, and 12. Three sources and their respective contribution rates for each HM were identified using CA, TEF, PCA, and PMF. The natural source contributed the most in relation to As (63.11%), Cr (61.67%), and Ni (70.80%); the source of fossil fuel combustion and atmospheric deposition contributed the most for Hg (97.0%); and the traffic source contributed the most for Zn (72.80%), Pb (69.75%), Cu (65.36%), and Cd (53.08%). The combined use of multiple methods was proven to be effective for HM source apportionment in soil. The results from different methods could be verified against each other, and the results from PMF were clearer and more complete compared to those from other approaches. The HMs originating from anthropogenic sources need to be remediated or appropriately controlled, but no action is required for the HMs originating from natural sources. It is of great importance to fully consider the sources of pollutants during soil remediation in order to avoid over-remediation.

## Figures and Tables

**Table 1:** Descriptive statistics of soil HM concentrations (mg/kg).

Elements		As	Cd	Cr	Cu	Ni	Pb	Zn	Hg
Method Detective Level		0.50	0.01	0.10	0.10	0.10	0.10	0.50	0.01
Topsoil (n = 13)	Min	3.89	0.050	14.45	11.86	13.42	7.79	29.78	0.020
	Max	13.24	0.23	28.61	61.99	22.98	53.72	144.54	8.13
	Mean	7.52	0.090	19.40	23.74	16.86	20.60	54.16	0.91
	SD	2.45	0.060	4.50	16.02	2.63	13.98	33.84	2.19
	CV	0.33	0.71	0.23	0.67	0.16	0.68	0.62	2.42
	Background value	9.70	0.07	68.10	23.60	29.00	25.40	102.60	0.070
	Samples exceeding background value	15.38%	38.46%	-	30.77%	-	23.08%	7.69%	76.92%
	Maximum exceedance as a multiple of background level	0.36	2.15	-	1.63	-	1.12	0.41	116.88
Subsoil (n = 51)	Min	1.53	0.020	8.14	4.36	6.84	4.26	16.17	0.010
	Max	16.86	0.16	29.75	27.99	31.24	16.90	94.02	0.20
	Mean	5.94	0.060	16.66	11.98	15.41	8.03	33.19	0.040
	SD	2.60	0.030	5.40	6.07	6.30	3.15	14.62	0.030
	CV	0.44	0.53	0.32	0.51	0.41	0.39	0.44	0.83
	Background value	9.70	0.070	68.10	23.60	29.00	25.40	102.60	0.070
	Samples exceeding background value	7.84%	29.41%	-	5.88%	-	-	-	5.88%
	Maximum exceedance as a multiple of background level	0.74	1.12	-	0.19	-	-	-	1.96

**Table 2:** Correlation coefficient matrix of the HM concentrations in the topsoil.

Element	As	Cd	Cr	Cu	Ni	Pb	Zn	Hg
As	1.000	0.688 **	0.555 *	0.390	0.577 *	0.302	0.220	0.370
Cd	0.688 **	1.000	0.461	0.637 *	0.475	0.580 *	0.555 *	0.623 *
Cr	0.555 *	0.461	1.000	0.538	0.940 **	0.462	0.198	0.276
Cu	0.390	0.637 *	0.538	1.000	0.544	0.973 **	0.758 **	0.845 **
Ni	0.577 *	0.475	0.940 **	0.544	1.000	0.451	0.264	0.243
Pb	0.302	0.580 *	0.462	0.973 **	0.451	1.000	0.736 **	0.884 **
Zn	0.220	0.555 *	0.198	0.758 **	0.264	0.736 **	1.000	0.652 *
Hg	0.370	0.623 *	0.276	0.845 **	0.243	0.884 **	0.652 *	1.000

* means that the correlation is significant at the 0.05 level and ** means that the correlation is significant at the 0.01 level.

## Data Availability

Not applicable.

## Appendix A

**Table A1:** TEF values of the HMs in the soil.

	As	Cd	Cr	Cu	Ni	Pb	Zn	Hg
Min	0.72	0.71	0.81	0.78	0.77	0.87	0.78	0.92
Max	1.82	4.71	1.73	5.54	1.56	7.34	4.19	905.5
Mean	1.28	1.47	1.19	2.00	1.12	2.61	1.63	79.91
>2	0	15.4%	0	30.8%	0	53.8%	15.4%	76.9%

**Table A2:** Total Variance Explained.

Element	Initial Eigenvalues			Extraction Sums of Squared Loadings			Rotation Sums of Squared Loadings		
	Total	% of Variance	Cumulative %	Total	% of Variance	Cumulative %	Total	% of Variance	Cumulative %
As	6.26	62.64	62.64	6.26	62.64	62.64	4.42	44.22	44.22
Cd	0.98	9.79	92.07	1.96	19.65	82.28	3.81	38.06	82.29
Cr	0.47	4.68	96.75						
Cu	0.17	1.67	98.41						
Ni	0.094	0.94	99.36						
Pb	0.054	0.54	99.89						
Zn	0.0026	0.026	99.99						
Hg	0.0012	0.012	100.00						

**Table A3:** Component and rotated component matrices for heavy metal concentrations.

Element	Component Matrix		Rotated Component Matrix	
	F1	F2	F1	F2
As	0.435	0.476	0.183	0.112
Cd	0.913	0.121	0.606	0.548
Cr	0.899	−0.323	0.902	0.334
Cu	0.917	0.167	0.418	0.871
Ni	0.843	−0.421	0.93	0.255
Pb	0.808	0.346	0.199	0.918
Zn	0.843	0.039	0.398	0.87
Hg	0.42	0.667	0.049	0.174
